# Impaired Cellular Immunity in the Murine Neural Crest Conditional Deletion of *Endothelin Receptor-B* Model of Hirschsprung’s Disease

**DOI:** 10.1371/journal.pone.0128822

**Published:** 2015-06-10

**Authors:** Ankush Gosain, Amanda J. Barlow-Anacker, Chris S. Erickson, Joseph F. Pierre, Aaron F. Heneghan, Miles L. Epstein, Kenneth A. Kudsk

**Affiliations:** 1 Department of Surgery, University of Wisconsin-Madison School of Medicine and Public Health, Madison, Wisconsin, United States of America; 2 Department of Neuroscience, University of Wisconsin-Madison School of Medicine and Public Health, Madison, Wisconsin, United States of America; 3 Veteran Administration Surgical Service, William S. Middleton Memorial Veterans Hospital, Madison, United States of America; Childrens Hospital Los Angeles, UNITED STATES

## Abstract

Hirschsprung’s disease (HSCR) is characterized by aganglionosis from failure of neural crest cell (NCC) migration to the distal hindgut. Up to 40% of HSCR patients suffer Hirschsprung’s-associated enterocolitis (HAEC), with an incidence that is unchanged from the pre-operative to the post-operative state. Recent reports indicate that signaling pathways involved in NCC migration may also be involved in the development of secondary lymphoid organs. We hypothesize that gastrointestinal (GI) mucosal immune defects occur in HSCR that may contribute to enterocolitis. *EdnrB* was deleted from the neural crest (*EdnrB* **^NCC-/-^**) resulting in mutants with defective NCC migration, distal colonic aganglionosis and the development of enterocolitis. The mucosal immune apparatus of these mice was interrogated at post-natal day (P) 21–24, prior to histological signs of enterocolitis. We found that *EdnrB* **^NCC-/-^** display lymphopenia of their Peyer’s Patches, the major inductive site of GI mucosal immunity. *EdnrB* **^NCC-/-^** Peyer’s Patches demonstrate decreased B-lymphocytes, specifically IgM^+^IgD^hi^ (Mature) B-lymphocytes, which are normally activated and produce IgA following antigen presentation. *EdnrB* **^NCC-/-^** animals demonstrate decreased small intestinal secretory IgA, but unchanged nasal and bronchial airway secretory IgA, indicating a gut-specific defect in IgA production or secretion. In the spleen, which is the primary source of IgA-producing Mature B-lymphocytes, *EdnrB* **^NCC-/-^** animals display decreased B-lymphocytes, but an increase in Mature B-lymphocytes. *EdnrB* **^NCC-/-^** spleens are also small and show altered architecture, with decreased red pulp and a paucity of B-lymphocytes in the germinal centers and marginal zone. Taken together, these findings suggest impaired GI mucosal immunity in *EdnrB* **^NCC-/-^** animals, with the spleen as a potential site of the defect. These findings build upon the growing body of literature that suggests that intestinal defects in HSCR are not restricted to the aganglionic colon but extend proximally, even into the ganglionated small intestine and immune cells.

## Introduction

Hirschsprung’s disease (HSCR, Online Mendelian Inheritence in Man #142623) is a common cause of intestinal obstruction in the newborn and is characterized by an absence of enteric nervous system (ENS) ganglion cells in the distal hindgut, extending from the rectum to a variable distance proximally. Failure of cranial-caudal neural crest cell (NCC) migration results in an aganglionic segment that exhibits tonic contraction, causing a functional bowel obstruction [1,2]. Untreated, this leads to progressive bowel distention, the development of Hirschsprung’s-associated enterocolitis (HAEC), and death. HAEC is a significant and potentially life-threatening complication of HSCR [3,4], with an incidence that ranges from 20–58% and carries a mortality of 2.5–9%, accounting for the majority of deaths from HSCR [5,6]. While HSCR is traditionally treated with surgical resection of the aganglionic segment and “pull-through” of ganglionated bowel to the anus, the incidence of HAEC appears to be unchanged from the pre-operative to post-operative state in both animals and humans [7–9], suggesting bowel obstruction is not the sole etiology. However, the precise etiology of HAEC remains elusive.

HAEC occurs in two forms, pre-operative (or neonatal) and post-operative [10–12]. Factors thought to contribute to the pathogenesis in both forms include abnormal goblet cell function, decreased secretory immunoglobulin A (SIgA) secretion, and leukocyte dysfunction[3,4,13]. Additionally, multiple investigators have postulated that HAEC develops in the setting of distal bowel obstruction[14] and have demonstrated similar histopathologic changes in experimental models of bowel obstruction to those in HAEC-affected bowel, including polymorphonuclear cell infiltration and mucous retention within the intestinal crypts, and the development of crypt abscesses[15]. However, while animal models of distal bowel obstruction share findings of focal inflammation and the development of punctate ulcers, they do not demonstrate the excessive mucous production or crypt abscesses that are found in HAEC [16]. Additionally, the clinical observation of continued susceptibility to HAEC in the post-pull-through period argues against obstruction as the sole etiology. Furthermore, investigations into an infectious cause for HAEC have failed to identify a single, responsible organism [17,18]. However, recent studies in mouse models of HSCR/HAEC have identified broader changes in the intestinal microbiome that may contribute to HAEC[19,20].

Multiple genetic defects have been associated with HSCR and failure of NCC migration, most commonly mutations of *Rearranged during transfection* (*Ret*) and of *Endothelin receptor B* (*EdnrB*)[1,21–27]. *RET* encodes a transmembrane receptor kinase, that binds a complex of the ligand Glial cell Derived Neurotrophic Factor (GDNF) and its receptor GDNF Family Receptor α (GFRα), thereby stimulating NCC survival, proliferation, migration and differentiation[28]. *Ret* has additionally been identified as necessary for the development of Peyer’s patches (PP)[29], the primary inductive site for gastrointestinal (GI) host defense[30], providing further evidence for a potential developmental link between the ENS and GI mucosal immunity. Unfortunately, *RET* knockout animals exhibit a severe phenotype with NCC colonization limited to the esophagus and stomach and renal agenesis[31]. These animals die shortly after birth, making them unsuitable for the study of post-natal HAEC[31].

*EdnrB* and its ligand, endothelin 3 (*ET3*), regulate NCC proliferation, migration and differentiation[32,33]. Naturally occurring mutations of both *EdnrB* and *ET3* have been found in mice; the piebald lethal strain lacks *EdnrB* and the lethal spotted strain lacks *ET3*[34,35]. Experimental mutation of *EdnrB* results in aganglionosis of the distal hindgut, mimicking that seen in the piebald lethal and lethal spotted strains as well as the common clinical finding in HSCR patients[36,37]. We have developed and characterized mice with a conditional neural crest-specific deletion of *EdnrB* (*EdnrB* ^**NCC-/-**^)[37]. These mice exhibit distal colonic aganglionosis and develop HAEC, closely modeling human HSCR and HAEC[19,38,39]. We have recently described the time course of development of HAEC, alterations in the colonic microbiota, and impaired innate immune function in these animals[19].

Here, we used the NCC deletion of *EdnrB* (*EdnrB* ^**NCC-/-**^) model of HSCR and HAEC to systematically examine the GI mucosal immune apparatus and its potential contribution to the development of HAEC. Prior to histological signs of enterocolitis, we find that *EdnrB* ^**NCC-/-**^ animals demonstrate a gut-specific deficiency in secretory IgA. These mice have small, lymphopenic PP with reduced mature B-lymphocytes. Interestingly, they also show increased mature B-lymphocytes in the spleen, the primary source of this cell population. Our findings suggest impaired cellular immunity may contribute to the development of HAEC.

## Materials and Methods

### Ethics Statement

All procedures were approved by the University of Wisconsin Institutional Animal Care and Use Committee (Protocol #M01392).

### Animals

We utilized a mouse model with NCC deletion of Endothelin Receptor B (*EdnrB* ^*flex3/flex3*^)[37]. Mating *TgWnt1-Cre/+;EdnrB* ^*flex3/+*^ mice with *Rosa26* ^*floxStop/tdTomato*^;*EdnrB* ^*flex3/flex3*^ mice resulted in either heterozygous (*EdnrB* ^*flex3/+*^ *)* or homozygous deletion of *EdnrB* (*EdnrB* ^*flex3/flex3*^ *)*, (defined throughout the manuscript as *EdnrB* ^**NCC+/-**^ and *EdnrB* ^**NCC-/-**^, respectively). In addition, NCC in these mice express tdTomato [39]. Mice were housed in a non-sterile environment and were allowed *ad libitum* access to standard rodent chow and water. *EdnrB* ^**NCC+/-**^ animals were utilized as littermate controls. *EdnrB* ^**NCC+/-**^ mice are available from Jackson Laboratories (Stock Number: 009063).

### Immunohistochemistry and Scoring for Enterocolitis

Tissue sections were either fixed in 4% formalin, processed (Tissue-Tek V.I.P), and embedded in paraffin or fixed in 4% paraformaldehyde and processed into 30% sucrose prior to sectioning. Segments of colon and ileum were harvested from *EdnrB* ^**NCC+/-**^ and *EdnrB* ^**NCC-/-**^ animals at P21-24 and P26-29 (n = 4–6 animals per group, per time point). Fresh specimens were fixed in 4% paraformaldehyde for 24 hours and submitted to the University of Wisconsin Department of Surgery Histology Core for further processing into paraffin blocks. Paraffin-embedded small intestine and colon specimens were sectioned at a thickness of 5 microns, deparaffinized in xylene and stained with routine hematoxylin & eosin (H&E), as previously described [19]. H&E-stained sections were scored for severity and depth of inflammation by blinded observers from the Department of Pathology using the semi-quantitative Murine Enterocolitis Grading System developed by Cheng, et.al.[40].

Paraffin tissue sections of PP were stained for T cells (CD3, M7254, 1:100, Rabbit anti-human polyclonal antibody, Dako) and B cells (CD45R/B220, 1:50, Rat anti-mouse polyclonal antibody, cat. 550286 BD Biosystems, San Jose, CA) and visualized with goat anti-rat IgG secondary antibody (Alexa Fluor 488, 1:500, Invitrogen, Carlsbad, CA) and goat anti-rabbit IgG secondary antibody (Alexa Fluor 568, 1:500, Invitrogen), respectively. Sections were counter-stained with DAPI solution (P36935, Invitrogen). 16μm cryosections of spleen were stained in a similar manner with CD3, B220 and MOMA (1:400, anti-rabbit biotin, ab51813, Abcam) and visualized with goat anti-rabbit Alexa Fluor 647, goat anti-rat Alexa Fluor 488 and streptavidin conjugated 568 (Invitrogen), respectively.

Slides were imaged on a Nikon A1 confocal microscope. Z-series were captured, processed, and analyzed with Nikon Elements (Nikon, Melville, NY, USA). The brightness and contrast may have been adjusted using Photoshop (Adobe systems, New York, NY).

### Isolation of Lymphocytes

After euthanasia, 500μL of blood was collected by cardiac puncture. Blood samples were diluted to 4mL with HBSS (Lonza, Walkersville, MD) and Ficoll-Paque (GE Healthcare, Precataway, NJ) separation used to obtain the lymphocyte layer.

The peritoneal cavity was opened and the small intestine (SI) isolated and flushed with 10mL of cold CMF-HBSS containing 50000U penicillin and 50mg/mL streptomycin (Sigma, St. Louis, MO). PP were removed from the entire length of the SI and placed into 1.5mL tubes of CMF-HBSS. PP were strained through 100μm mesh with a total volume of 15mL CMF-HBSS. The effluent was collected and spun at 1000rpm at 4°C for 10 minutes. Supernatant was removed and the pellet re-suspended in 2mL of 1xPBS containing 0.5% bovine serum albumin (BSA) and stored on ice.

The spleen was removed from the peritoneal cavity and strained through a 40μm nylon mesh filter. The cell suspension was transferred to a 15mL tube which was spun at 1000rpm for 10 minutes at 4°C. The supernatant was discarded and the pellet was re-suspended in 5mL of 1X Lysis buffer (PharM Lyse, Becton-Dickinson, Franklin Lakes, NJ). This was incubated for 15 minutes at room temperature (RT) protected from light. The tube was then spun at 1000rpm for 10 minutes at 4°C. The supernatant was discarded and the pellet re-suspended with 2mL 1xPBS containing 0.5% BSA.

To isolate bone marrow lymphocytes, the femur was removed and the tips excised. The bone was flushed with CMF-HBSS via syringe with a 27 gauge needle and bone marrow cells filtered through a 40μm nylon mesh filter. Red blood cells were removed by incubation with Lysis buffer, as described above.

Cells were counted by Trypan blue exclusion on a hemocytometer and recorded. For flow cytometry, the cell density was adjusted to approximately 1x10^6^ cells/mL with staining buffer (Becton-Dickinson, Franklin Lakes, NJ) prior to antibody staining.

### Flow Cytometry Analysis

75μL of the cell solution (1x10^6^ cells/mL) was added to a clean 15mL tube (Falcon 2058) in duplicate with 10μL of respective antibody solution (T or B cell antibody solution). The T cell antibody solution contained the following fluorochrome conjugated antibodies from BD Pharmingen (San Diego, CA) at a final concentration of 2.5μg/mL in staining buffer: Pacific Blue-conjugated mAb to CD3 (145-2C11), Alexa 700-conjugated mAb to CD4 (RM4-5), PerCP-Cy5.5-conjugated mAb to CD8a (53–6.7), APC-Alexa fluor 750 mAB to L-selectin (Ly-22). The B cell antibody solution contained the following fluorochrome-conjugated antibodies from BD Pharmingen at a final concentration of 2.5μg/mL: Pacific Blue-conjugated mAb to CD3 (145-2C11), Alexa 700-conjugated mAb to CD45R/B220 (RA3-6B2), PerCP-Cy5.5-conjugated mAb to IgM (R6-60.2), and FITC-conjugated mAb to IgD (11-26c.2a), PE-Cy7 mAB to L-selectin (Ly-22). After addition of the antibody solution, the cells were incubated for 20 minutes on ice protected from light. 1mL of staining buffer was added to each tube and tubes were spun at 1000rpm for 10 minutes at 5°C. The supernatant was then aspirated and 1mL of staining buffer added to wash. This step was repeated and tubes were then stored at 4°C protected from light.

The BD LSR II Flow cell cytometer at the Wisconsin Institute of Medical Research was used for sample analysis. FlowJo (Ashland, OR) was used to analyze the raw data.

### Bronchoalveolar Lavage, Nasal Airway Lavage and Small Intestinal Lavage and IgA Antibody Quantitative Analysis

After washing the skin with ethanol, a midline incision was made over the ventral neck slightly superior to the thoracic inlet to allow access to the trachea. A tracheotomy was then created and an 18g catheter attached to a tuberculin syringe was inserted. 1mL of saline was injected slowly into the lungs and collected as bronchoalveolar lavage (BAL) fluid. The same tracheotomy site was used to inject 1mL of saline cephalad and fluid exiting the nose was collected as nasal airway lavage (NAL). Aliquots were frozen at -80°C. The small intestine was removed and the lumen rinsed with 10mL Hank’s Balanced Saline Solution (HBSS; Bio Whitaker, Walkersville, MD). The luminal rinse (small intestinal lavage, SIL) was centrifuged at 2000x*g* for 10 minutes and supernatant aliquots were frozen at -80°C.

IgA concentration from the NAL, BAL and SIL fluid was measured by sandwich enzyme-linked immunosorbent assay (ELISA), as previously described [Sano 2009]. Fluid IgA concentrations were calculated by plotting their absorbance values on the IgA standard curve, which was calculated using a 4-parameter logistic fit with SOFTmax PRO software (Molecular Device, Sunnyvale, CA).

### Statistical analysis

The data are expressed as means ± standard error of the mean. Statistical significance was determined using Student’s *t*-test. Differences were considered to be statistically significant at *p<0*.*05*. Statistical calculations were performed with Microsoft Excel (Microsoft, Redmond, WA) or StatView (SAS, Cary, NC).

## Results

### *EdnrB* ^NCC-/-^ animals develop intestinal inflammation in the fourth week of life

**Fig 1:**
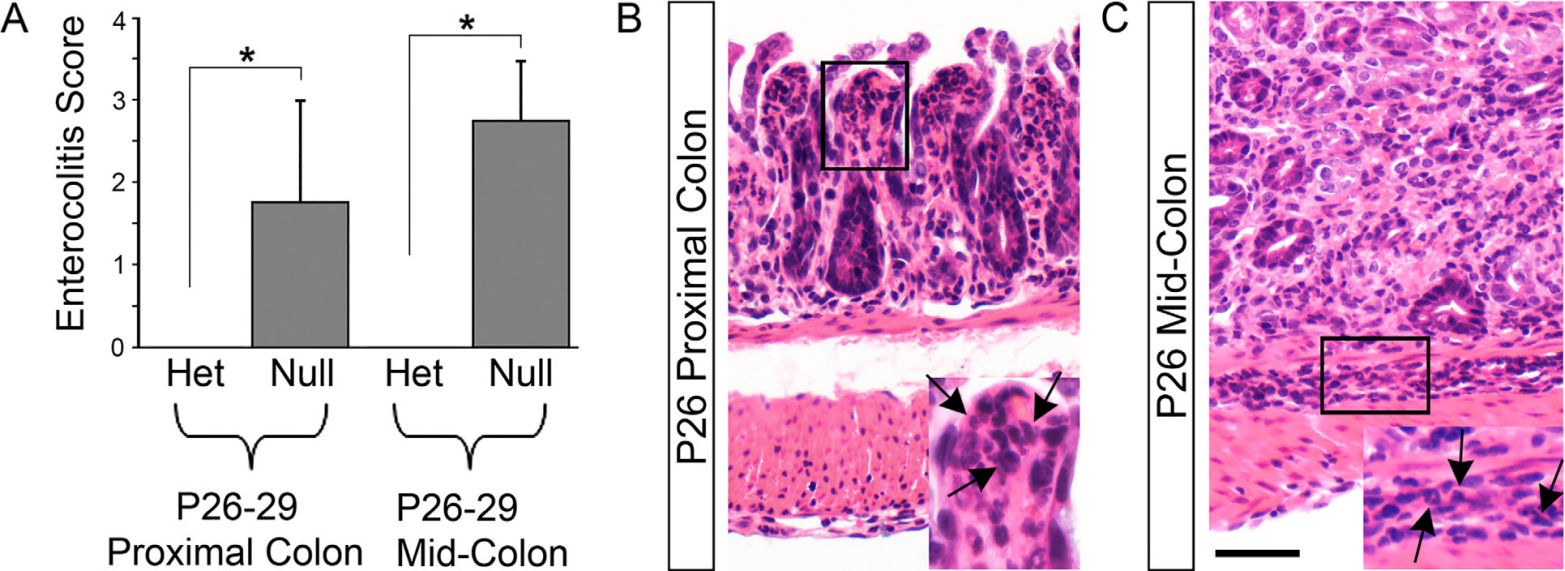
*EdnrB* ^NCC-/-^ animals develop intestinal inflammation in the fourth week of life. Inflammation within proximal colon and mid-colon of P26-29 *EdnrB* ^**NCC+/-**^ (Het) and *EdnrB* ^**NCC-/-**^ (Null) animals was graded using the previously published Murine Enterocolitis Grading System (Cheng, et.al., 2010). (A) There was evidence of inflammation in both the (B) proximal and (C) mid-colon of the *EdnrB* ^**NCC-/-**^ animals at the later time point. Arrows indicate inflammatory cells. Scale bar = 50 μm. (*p<0.05).

The piebald lethal mouse strain, which contains a naturally-occurring *EdnrB* mutation, and conventional *EdnrB* knockout mice experience mortality near the fourth postnatal (P) week [40,41]. We have previously confirmed a similar timing of mortality in our model of NCC conditional deletion of *EdnrB*, with *EdnrB* ^**NCC-/-**^ experiencing 50% mortality by P26 and 90% mortality by P32 [19]. To correlate histological evidence of colonic inflammation with this time course, we examined the distal small intestine, proximal colon and mid-colon of P21-24 and P26-29 *EdnrB* ^**NCC+/-**^ and *EdnrB* ^**NCC-/-**^ animals using the previously published Murine Enterocolitis Grading System [40] (Table 1 and Fig 1). There was evidence of inflammation in both the proximal and mid-colon of the *EdnrB* ^**NCC-/-**^ animals at P26-29. However, no inflammation was seen in the *EdnrB* ^**NCC-/-**^ animals at the early time point or in the small intestine at the later time point. Finally, no inflammation was seen in the *EdnrB* ^**NCC+/-**^ animals at either time point or anatomic location.

**Table 1:** Enterocolitis Score by Anatomic Location.

	*EdnrB* ^NCC+/-^ (n = 6)	*EdnrB* ^NCC-/-^ (n = 4)	*p* value
**Proximal Colon**			
Depth	0	1.0 ± 0.7	0.08
Severity	0	0.75 ± 0.5	0.06
Total	0	1.75 ± 1.2	0.07
**Mid-Colon**			
Depth	0	1.25 ± 0.25	0.0002 (*)
Severity	0	1.5 ± 0.5	0.005 (*)
Total	0	2.75 ± 0.75	0.002 (*)

The distal small intestine, proximal colon and mid-colon of P21-24 and P26-29 *EdnrB* ^**NCC+/-**^ and *EdnrB* ^**NCC-/-**^ animals were analyzed using the previously published Murine Enterocolitis Grading System. No inflammation was seen at the P21-24 time point or in the distal small intestine in either group. Data are presented for proximal colon and mid-colon at P26-29, showing depth of inflammation and severity of inflammation on an ordinal scale.

Statistically significant *p* values are indicated by an *.

### Peyer’s Patches of *EdnrB* ^NCC-/-^ animals are small in size but have normal architecture

**Fig 2:**
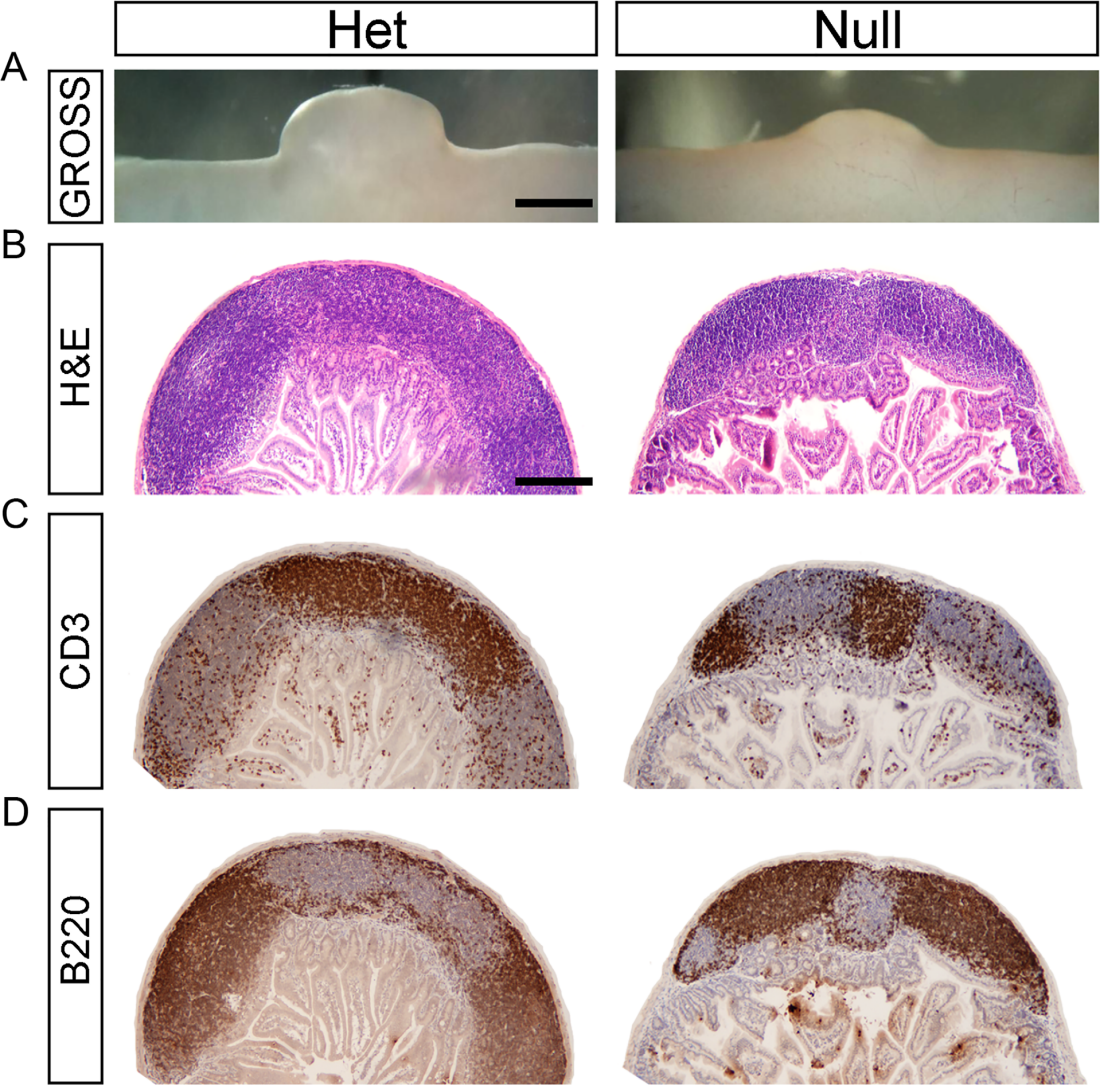
Peyer’s Patches of *EdnrB* ^NCC-/-^ animals are small in size but have normal architecture. (A) Gross photographs of representative PP isolated from *EdnrB* ^**NCC+/-**^ and *EdnrB* ^**NCC-/-**^ animals demonstrating the size difference. Scale bar = 1000μm. (B) H&E staining of PP cross-sections from *EdnrB* ^**NCC+/-**^ and *EdnrB* ^**NCC-/-**^ animals. Architectural features are preserved in *EdnrB* ^**NCC-/-**^. Scale bar = 400μm (applies to B,C,D). (C) IHC of PP cross-sections with CD3 labeling T cells. (D) IHC of PP cross-sections with B220 labeling B cells. The spatial distribution of B-cells and T-cells is similar between *EdnrB* ^**NCC+/-**^ and *EdnrB* ^**NCC-/-**^.

PP are distinct collections of immune cells located along the anti-mesenteric surface of the bowel; they display follicular architecture similar to lymph nodes and are the primary inductive site for gut mucosal immunity [42]. Previous observations that PP are absent in *RET* ^-/-^ animals and are diminished in *RET* ^51/51^ hypomorphic animals [29] prompted us to investigate whether the *EdnrB* ^**NCC-/-**^ animals exhibit alterations in their GI immune apparatus. We harvested small bowel from *EdnrB* ^**NCC-/-**^ and *EdnrB* ^**NCC+/-**^ at P21-24 [19]. We found that the PP of *EdnrB* ^**NCC-/-**^ animals are smaller than those of *EdnrB* ^**NCC+/-**^ animals on gross examination (Fig 2). To quantify this, cross-sectional measurements of PP height and width were taken and area calculated. We found that *EdnrB* ^**NCC-/-**^ PP have significantly smaller cross-sectional area than *EdnrB* ^**NCC+/-**^ (*EdnrB* ^**NCC+/-**^ 1.15±0.05 mm^2^ vs. *EdnrB* ^**NCC-/-**^ 0.49±0.04 mm^2^, *p* = 4.1x10^-17^). Although smaller in size, H&E staining demonstrates that the architecture of *EdnrB* ^**NCC-/-**^ PP is preserved, with distinct germinal centers, subepithelial domes and follicle-associated epithelium (Fig 2). Additionally, while the extent of B220+ and CD3+ staining is decreased in the *EdnrB* ^**NCC-/-**^ animals, suggesting fewer cells, the distribution of B220+ B-lymphocytes and CD3+ T-lymphocytes in distinct B- and T-cell zones within the PP appears unchanged between *EdnrB* ^**NCC+/-**^ and *EdnrB* ^**NCC-/-**^ animals (Fig 2). Finally, unlike the observation in *RET* mutant models, we did not observe a difference in the number of PP between groups (*EdnrB* ^**NCC+/-**^ 9.75±0.85 vs. *EdnrB* ^**NCC-/-**^ 7.25±1.3, *p* = 0.16).

### Peyer’s Patches of *EdnrB* ^NCC-/-^ animals exhibit B-cell lymphopenia

**Fig 3:**
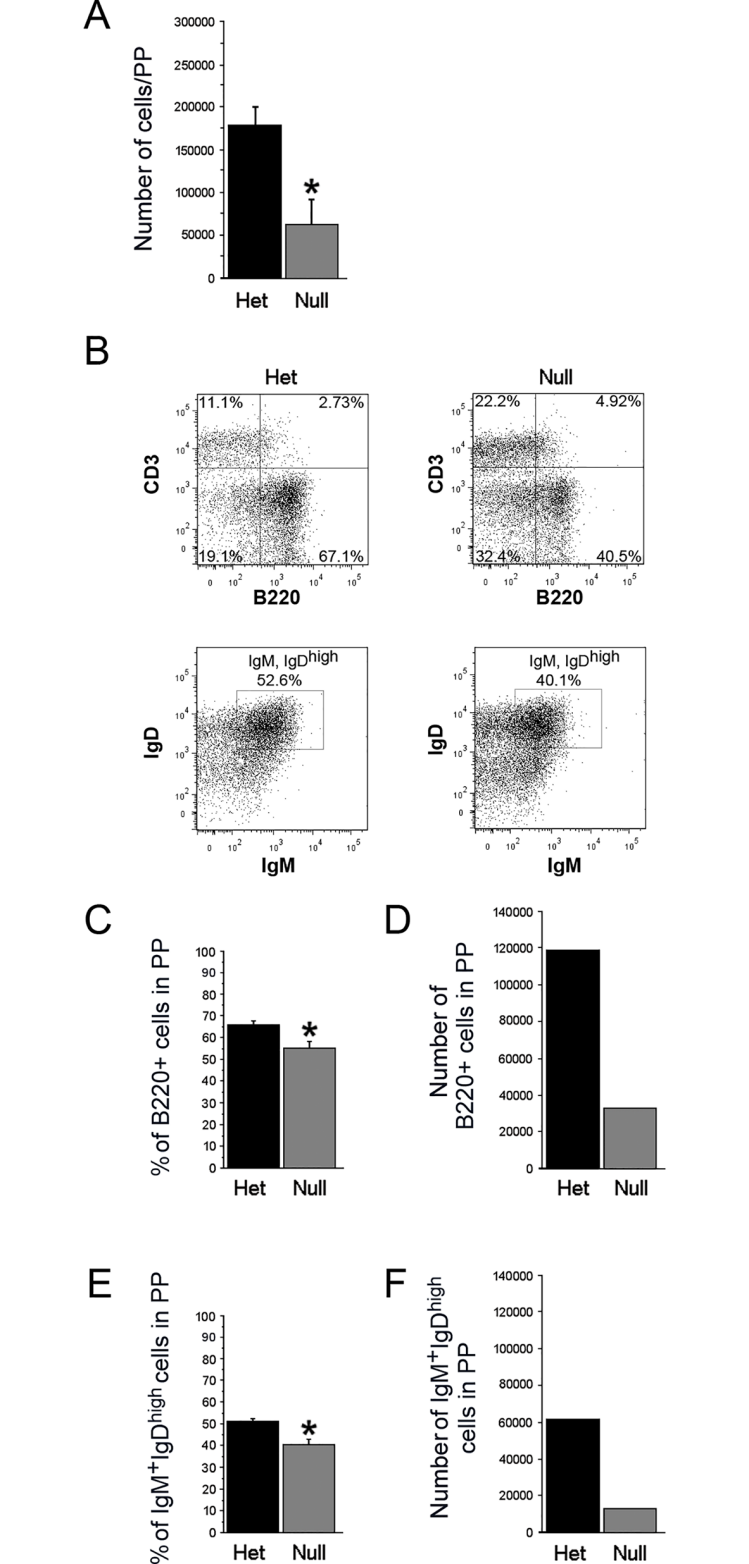
Peyer’s Patches of *EdnrB* ^NCC-/-^ animals exhibit B-cell lymphopenia. (A) Total lymphocytes (per PP) are decreased in *EdnrB* ^**NCC-/-**^ vs. *EdnrB* ^**NCC+/-**^ (*p = 0.0196). (B) Representative flow cytometry gating of PP showing CD3 and B220 to identify T- and B-lymphocytes (top) and IgD and IgM to identify B220^+^IgM^+^IgD^high^ (mature) B-lymphocytes (bottom). (C) The proportion of B-lymphocytes (B220+) per PP is decreased in *EdnrB* ^**NCC-/-**^ vs. *EdnrB* ^**NCC+/-**^ (*p = 0.01). (D) The number of B-lymphocytes per PP is decreased in *EdnrB* ^**NCC-/-**^ vs *EdnrB* ^**NCC+/-**^ (calculated values). (E) The proportion of B220^+^IgM^+^IgD^high^ mature B-lymphocytes is decreased in *EdnrB* ^**NCC-/-**^ vs.—het PP (*p = 0.015). (F) The number of mature B-lymphocytes per PP is decreased in *EdnrB* ^**NCC-/-**^ vs *EdnrB* ^**NCC+/-**^ (calculated values).

Given their small size, we next examined the cellular composition of PP in the *EdnrB* ^**NCC-/-**^ animals. We isolated PP from *EdnrB* ^**NCC+/-**^ and *EdnrB* ^**NCC-/-**^ animals and employed mechanical dispersion to isolate lymphocytes. *EdnrB* ^**NCC-/-**^ PP were hypocellular (*EdnrB* ^**NCC+/-**^ 18x10^4^±2x10^4^ vs. *EdnrB* ^**NCC-/-**^ 6x10^4^± 3x10^4^, *p* = 0.0196, n = 6, Fig 3), which was expected given the smaller size as compared to *EdnrB* ^**NCC+/-**^ PP. We then performed flow cytometry, using CD3+ to identify total T-lymphocytes and B220+ to identify total B-lymphocytes (Fig 3). The proportion of total T-lymphocytes was unchanged between the two groups (*EdnrB* ^**NCC+/-**^ 19.3±0.9% vs. *EdnrB* ^**NCC-/-**^ 18.9±2.1%, *p* = 0.88, n = 6, S1 Fig and Table 2). Additionally, we did not see any differences in the proportions of CD3+CD4+ or CD3+CD8+ T-lymphocytes between the two groups (S1 Fig and Table 2). In contrast, the proportion of total B-lymphocytes in *EdnrB* ^**NCC-/-**^ PP was significantly decreased to ~80% of the level seen in the *EdnrB* ^**NCC+/-**^ PP (*EdnrB* ^**NCC+/-**^ 66.2±1.6% vs. *EdnrB* ^**NCC-/-**^ 55.4±3.6%, *p* = 0.01, Fig 3), representing a 3.6-fold reduction in the number of PP B cells (*EdnrB* ^**NCC+/-**^ 119160 vs. *EdnrB* ^**NCC-/-**^ 33240, Fig 3 and Table 2).

**Table 2:** Summary results of cellularity findings in *EdnrB* ^NCC+/-^ and *EdnrB* ^NCC-/-^ Peyer’s Patches (PP), Spleen (SPL) and Peripheral Blood (PBL).

**Peyer's Patches (PP)**	*EdnrB* ^NCC+/-^	*EdnrB* ^NCC-/-^	***p***
# of PP	9.75 ± 0.85	7.25 ± 1.3	0.16
# cells per PP	18x104 ± 2x104	6x104 ± 3x104	0.0196 (*)
% B cells	66.2 ± 1.6%	55.4 ± 3.6%	0.01 (*)
Calculated absolute # of B cells per PP	119160	33240	*(calculated)*
% Mature B cells	51.7 ± 1.0%	40.4 ± 2.3%	0.015 (*)
Calculated absolute # of Mature B cells per PP	61605	13429	*(calculated)*
% T cells	19.3 ± 0.9%	18.9 ± 2.1%	NS
Calculated absolute # of T cells per PP	34740	11280	*(calculated)*
% CD4+ T cells	65.9 ± 2.0%	59.4 ± 5.9%	NS
Calculated absolute # of CD4+ T cells per PP	22893	6700	*(calculated)*
% CD8+ T cells	23.2 ± 1.5%	25.1 ± 2.5%	NS
Calculated absolute # of CD8+ T cells per PP	8060	2831	*(calculated)*
**Spleen (SPL)**	***EdnrB*^NCC+/-^**	***EdnrB*^NCC-/-^**	***p***
# cells per SPL	2.6x107 ± 0.19x107	1.2x107 ± 0.19x107	0.002 (*)
% B cells	60.0 ± 1.1%	43.4 ± 2.8%	0.000053 (*)
Calculated absolute # of B cells per SPL	15600000	5208000	*(calculated)*
% Mature B cells	32.9 ± 1.0%	39.1 ± 2.0%	0.00023 (*)
Calculated absolute # of Mature B cells per SPL	5132400	2036328	*(calculated)*
% T cells	15.4 ± 0.7%	35.5 ± 3.5%	0.000042 (*)
Calculated absolute # of T cells per SPL	4004000	4260000	*(calculated)*
**Peripheral Blood (PBL)**	***EdnrB*^NCC+/-^**	***EdnrB*^NCC-/-^**	***p***
% L-selectin(+) Mature B cells	35 ± 2.7%	38.4 ± 4.7%	NS
Calculated absolute # of L-selectin(+) Mature B cells/microliter	52399	46591	*(calculated)*

Rows with a p value displayed were measured experimentally (see results for details). Rows with “calculated” listed under the p value are computed based on other measured values.

Statistically significant *p* values are indicated by an *.

### *EdnrB* ^NCC-/-^ animals demonstrate a gut-specific SIgA deficiency

**Fig 4:**
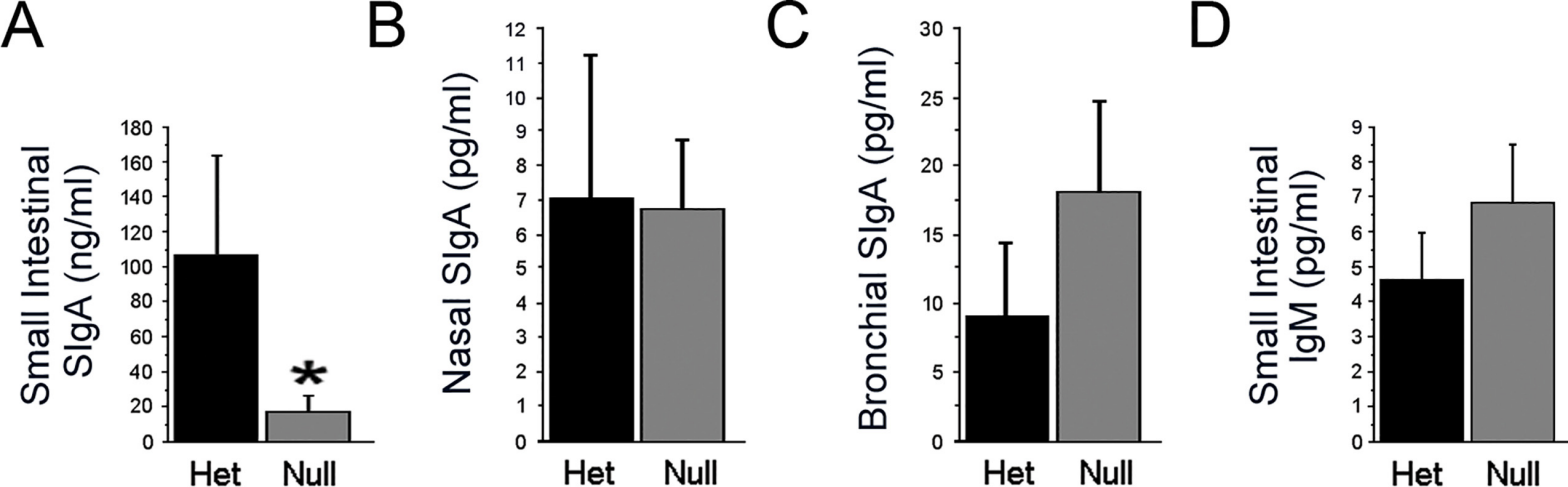
*EdnrB* ^NCC-/-^ animals demonstrate a gut-specific deficiency of Secretory IgA (SIgA). Secretory IgA was measured by ELISA in (A) small intestinal lavage fluid (*p = 0.002), (B) nasal airway lavage fluid, and (C) bronchoalveolar lavage fluid of *EdnrB* ^**NCC+/-**^ and *EdnrB* ^**NCC-/-**^ animals. (D) Additionally, IgM in small intestinal lavage was measured.

B-lymphocytes in the PP are activated by antigen presentation to become IgA-producing plasma cells. These activated B-lymphocytes circulate and home to the lamina propria where they produce and secrete IgA. Secretory IgA (SIgA) is the principle effector of antigen-specific immune defense along the mucosal surface[43]. *EdnrB* ^**NCC-/-**^ animals display clinical signs of enterocolitis (lethargy, ruffled coat, decreased oral intake) around P26 with a median survival of 29 days[19]. To determine if the B-lymphocytopenia observed at P21-24 is associated with impaired functional immunity, we performed nasal airway lavage, bronchoaleveolar lavage and small intestinal lavage on *EdnrB* ^**NCC+/-**^ and *EdnrB* ^**NCC-/-**^ animals at P26 and measured SIgA by ELISA. SIgA was markedly decreased in the small intestine of *EdnrB* ^**NCC-/-**^ animals compared to *EdnrB* ^**NCC+/-**^ (*EdnrB* ^**NCC+/-**^ 106.5±56.7ng/mL vs. *EdnrB* ^**NCC-/-**^ 18±9.8ng/mL, *p* = 0.002, n = 6, Fig 4). No differences were seen in SIgA levels in either the nasal airway lavage (*EdnrB* ^**NCC+/-**^ 7.1±4.2pg/mL vs. *EdnrB* ^**NCC-/-**^ 6.8±2pg/mL, *p* = 0.95, n = 8, Fig 4) or bronchoalveolar lavage (*EdnrB* ^**NCC+/-**^ 9.1±5.4pg/mL vs. *EdnrB* ^**NCC-/-**^ 18±6.6pg/mL, *p* = 0.31, n = 8, Fig 4). Previous investigations in humans have demonstrated increased levels of IgM in addition to alterations in IgA in the bowel during enterocolitis[44]. To evaluate this possibility in the *EdnrB* ^**NCC-/-**^ animals, we measured IgM levels in the small intestine lavage fluid. We did not detect differences in small bowel luminal IgM between *EdnrB* ^**NCC+/-**^ and *EdnrB* ^**NCC-/-**^ animals (*EdnrB* ^**NCC+/-**^ 4.6±1.4pg/mL vs. *EdnrB* ^**NCC-/-**^ 6.8±1.7pg/mL, *p* = 0.3, n = 8, Fig 4).

### Mature B-lymphocytes are reduced in *EdnrB* ^NCC-/-^ PP and enriched in the spleen

A specific subset (IgM^+^IgD^high^) of B-lymphocytes, termed mature B-lymphocytes, in the PP are responsible for the majority of murine intestinal IgA production [45]. We re-examined P21-24 PP lymphocytes by flow cytometry (Fig 3) and found that the mature B-lymphocyte population is decreased in *EdnrB* ^**NCC-/-**^ animals (*EdnrB* ^**NCC+/-**^ 51.7±1% vs. *EdnrB* ^**NCC-/-**^ 40.4±2.3%, *p* = 0.015, n = 6, Fig 3). This relationship held true for numbers of PP mature B-lymphocytes as well (*EdnrB* ^**NCC+/-**^ 61605 vs. *EdnrB* ^**NCC-/-**^ 13429, Fig 3).

**Fig 5:**
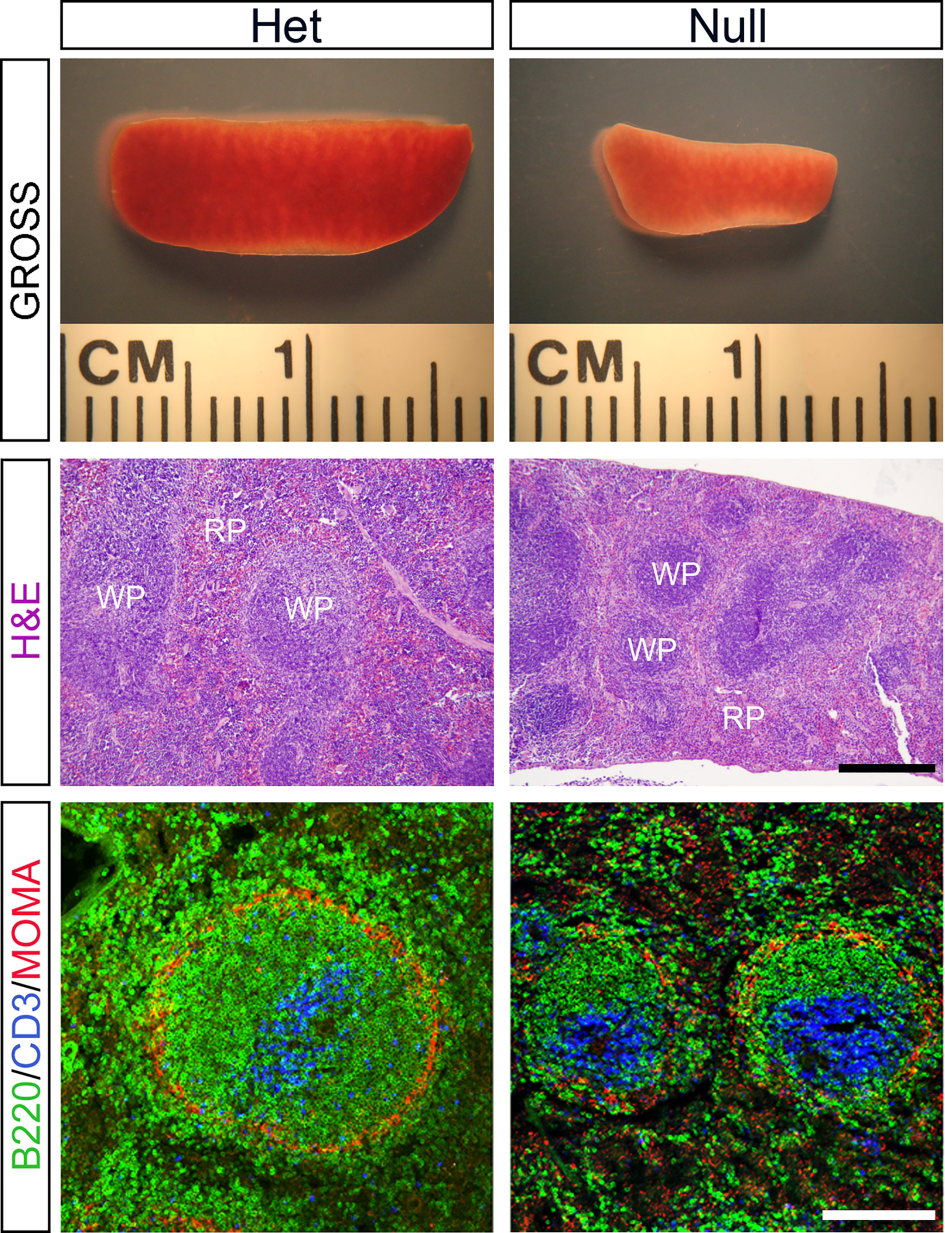
The spleen of *EdnrB* ^NCC-/-^ animals is small in size and has abnormal architecture. (A) Gross photographs of representative spleens isolated from *EdnrB* ^**NCC+/-**^ and *EdnrB* ^**NCC-/-**^ animals demonstrating the size difference. (B) H&E staining of spleen cross-sections from *EdnrB* ^**NCC+/-**^ and *EdnrB* ^**NCC-/-**^ animals. *EdnrB* ^**NCC-/-**^ spleens demonstrate decreased red pulp (RP) and white pulp (WP). Scale bar = 600μm. (C) IHC of spleen cross-sections with B220 (green) and CD3 (blue). There are decreased B-cells in the germinal centers and a paucity of these cells within the marginal zone, located outside the ring of MOMA+ (red) staining. Scale bar = 200μm.

**Fig 6:**
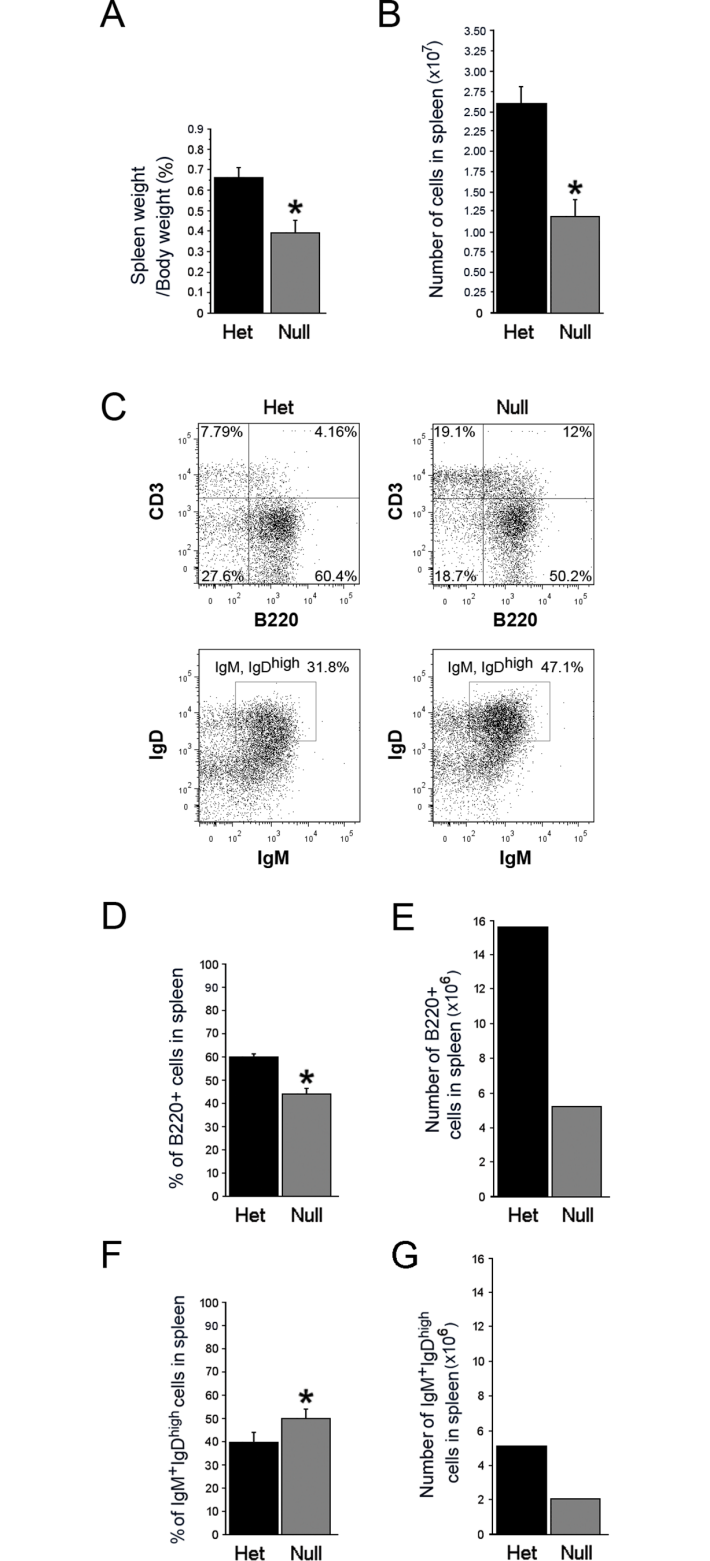
The spleen of *EdnrB* ^NCC-/-^ animals exhibits B-cell lymphopenia. (A) Organ weights of the spleen (*p = 0.02) as a proportion of body weight, indicating that the spleens of *EdnrB* ^**NCC-/-**^ animals are small for size. (B) Total lymphocytes (per spleen) are decreased in *EdnrB* ^**NCC-/-**^ vs. *EdnrB* ^**NCC+/-**^ (*p = 0.002). (C) Representative flow cytometry gating of splenocytes showing CD3 and B220 to identify T- and B-lymphocytes (top) and IgD and IgM to identify B220^+^IgM^+^IgD^high^ (mature) B-lymphocytes (bottom). (D) The proportion of B-lymphocytes (B220+) per spleen is decreased in *EdnrB* ^**NCC-/-**^ vs. *EdnrB* ^**NCC+/-**^ (*p = 0.000053). (E) The number of B-lymphocytes per spleen is decreased in *EdnrB* ^**NCC-/-**^ vs.—het spleens (calculated values). (F) B220^+^IgM^+^IgD^high^ mature B-lymphocytes are increased in *EdnrB* ^**NCC-/-**^ vs.—het spleen (*p = 0.00023). (G) The number of mature B-lymphocytes per spleen is decreased in *EdnrB* ^**NCC-/-**^ vs.—het spleens (calculated values).

In the mouse, mature B-lymphocytes in PP are primarily of splenic origin [45,46]. Initial observations by Cheng, et.al. indicated the presence of small spleens and splenic lymphopenia in the conventional *EdnrB* knockout model of HSCR [47], indicating that the immune system defects in this animal model may extend beyond the GI tract. We have extended their analysis, in our NCC-conditional deletion of *EdnrB* model, that the spleens in P21-24 *EdnrB* ^**NCC-/-**^ are smaller in size than those in the *EdnrB* ^**NCC+/-**^ (Fig 5). We found that the spleens of *EdnrB* ^**NCC-/-**^ weighed significantly less than those of the *EdnrB* ^**NCC+/-**^ as a proportion of the total body weight (*EdnrB* ^**NCC+/-**^ 0.67±0.05% vs. *EdnrB* ^**NCC-/-**^ 0.39±0.06%, *p* = 0.02, n = 10, Fig 6). We then examined splenic architecture and noted a decrease in the size of both the white and red pulp of the *EdnrB* ^**NCC-/-**^ spleen (Fig 5). We further examined architecture by immmunostaining (Fig 5) and observed a paucity of B-lymphocytes in the germinal centers of *EdnrB* ^**NCC-/-**^ spleens, and a marked decrease of B-lymphocytes within the marginal zones, suggesting potential defects in B-lymphocyte maturation [48].

Having noted alterations in splenic architecture and distribution of lymphocytes within the parenchyma of the spleen, we then determined total lymphocyte counts in the spleens (Fig 6). We observed splenic lymphopenia in the *EdnrB* ^**NCC-/-**^ animals (*EdnrB* ^**NCC+/-**^ 2.6x10^7^±0.19x10^7^ vs. *EdnrB* ^**NCC-/-**^ 1.2x10^7^±0.19x10^7^, *p* = 0.002), as has previously been described in the conventional *EdnrB* mutants [47]. In addition, because the spleen is the primary source of IgA-producing mature B-lymphocytes in the PP, we analyzed B-lymphocyte subsets (Fig 6 and Table 2). We observed a decrease in the proportion of total (B220^+^) B-lymphocytes in *EdnrB* ^**NCC-/-**^ (*EdnrB* ^**NCC+/-**^ 60±1.1% vs. *EdnrB* ^**NCC-/-**^ 43.4±2.8%, *p* = 0.000053, Fig 6) as well as in numbers of total B lymphocytes (*EdnrB* ^**NCC+/-**^ 15600000 vs. *EdnrB* ^**NCC-/-**^ 5208000, Fig 6). In contrast to our finding in *EdnrB* ^**NCC-/-**^ PP, we observed an increase in the proportion of mature B-lymphocytes in the *EdnrB* ^**NCC-/-**^ spleen (*EdnrB* ^**NCC+/-**^ 32.9±1% vs. *EdnrB* ^**NCC-/-**^ 39.1±2%, *p* = 0.00023, Fig 6), although the numbers were decreased (*EdnrB* ^**NCC+/-**^ 5132400 vs. *EdnrB* ^**NCC-/-**^ 2036328, Fig 6).

To determine if the both the PP and splenic B lymphopenia we observed in the *EdnrB* ^**NCC-/-**^ animals was secondary to suppression of bone marrow lymphopoiesis, we examined the pre/pro-B lymphocyte and mature B lymphocyte populations at P21. We noted no differences in either population at this time point (pre/pro B: *EdnrB* ^**NCC+/-**^ 70.5±1.3% vs. *EdnrB* ^**NCC-/-**^ 71.8±3.6%, *p* = 0.76; mature B: *EdnrB* ^**NCC+/-**^ 9.6±1.1% vs. *EdnrB* ^**NCC-/-**^ 10.4±2.8%, *p* = 0.81).

### L-selectin/MAdCAM-1 Mediated Trafficking of B-lymphocytes from Peripheral Blood to PP is preserved

**Fig 7:**
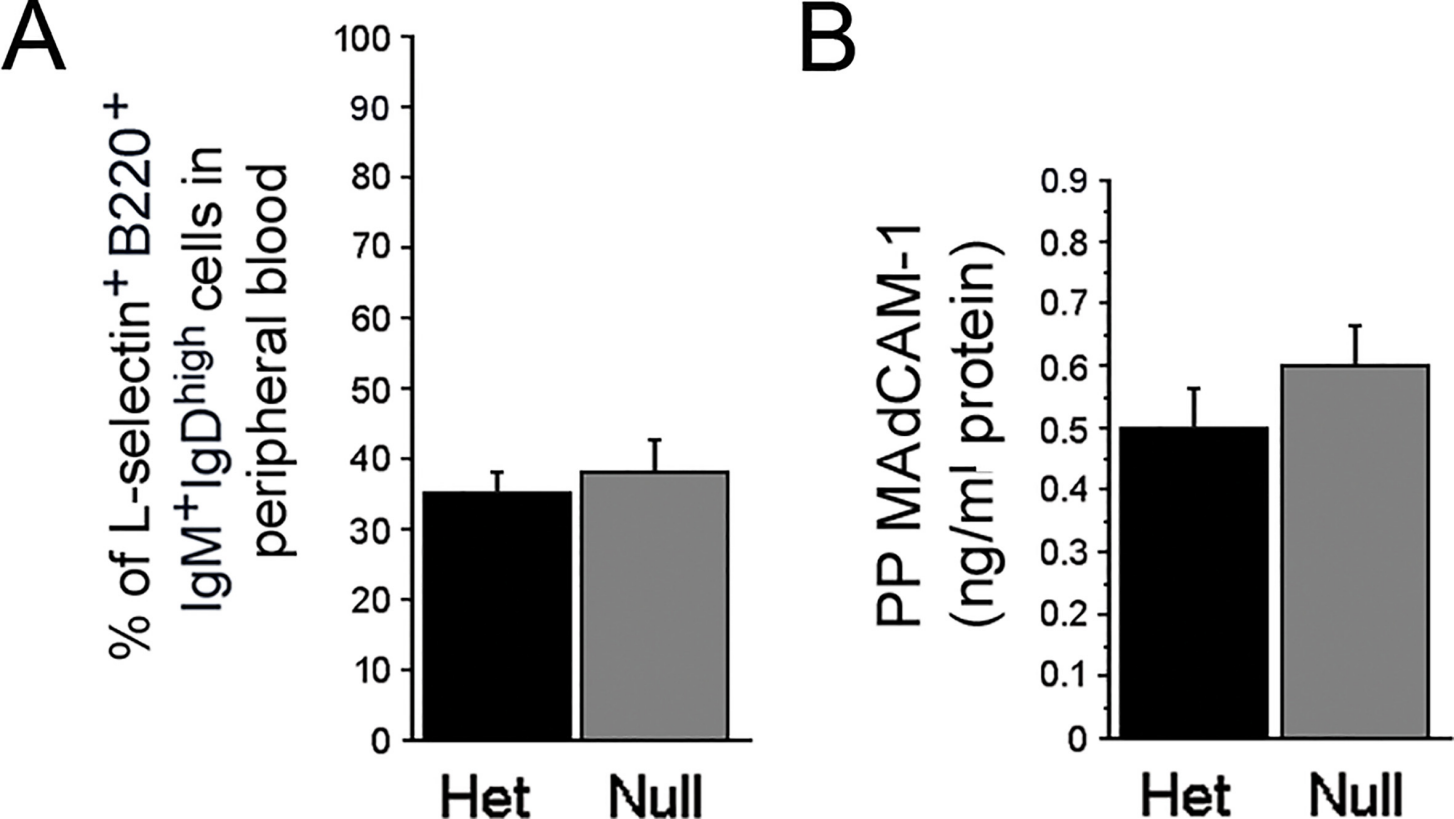
Trafficking of B-lymphocytes from Peripheral Blood to PP is preserved. (A) The proportion of circulating mature B-lymphocytes expressing L-selectin is unchanged between *EdnrB* ^**NCC+/-**^ and-null animals. (B) Levels of PP Mucosal Addressin Cell Adhesion Molecule-1 (MAdCAM-1) are unchanged between *EdnrB* ^**NCC+/-**^ and *EdnrB* ^**NCC-/-**^ animals.

B-lymphocytes targeted for mucosa-associated lymphoid tissue (e.g. PP) are identified by the cell-surface integrin L-selectin [49]. L-selectin expression is acquired by B-lymphocytes in the spleen as part of the process of normal maturation [50]. Mature B-lymphocytes expressing L-selectin (L-selectin^high^) leave the spleen, circulate in the peripheral blood, and home to the PP. To determine if the proportional increase in mature B-lymphocytes in the spleen and concurrent decrease in the PP of *EdnrB* ^**NCC-/-**^ was due to a failure of trafficking of circulating B-cells from peripheral blood to the PP, we measured the B220^+^IgM^+^IgD^high^L-selectin^high^ population in peripheral blood. We found no differences in the L-selectin^high^ mature B-lymphocyte populations between *EdnrB* ^**NCC+/-**^ and *EdnrB* ^**NCC-/-**^ animals (*EdnrB* ^**NCC+/-**^ 35±2.7% vs. *EdnrB* ^**NCC-/-**^ 38.4±4.7%, *p* = 0.6, n = 9, Fig 7 and Table 2).

L-selectin interacts with Mucosal Addressin Cell Adhesion Molecule-1 (MAdCAM-1) on the high endothelial venules of the PP to mediate attachment of the lymphocytes and localization to the PP [49]. To determine if there is a defect in homing of circulating mature B-lymphocytes to the PP, we measured MAdCAM-1 in PP at P21-24 by ELISA. We found similar levels of MAdCAM-1 in PP from *EdnrB* ^**NCC+/-**^ and *EdnrB* ^**NCC-/-**^ animals (*EdnrB* ^**NCC+/-**^ 0.5±0.06 ng/mg vs. *EdnrB* ^**NCC-/-**^ 0.6±0.06ng/mg, *p* = 0.2, n = 10, Fig 7).

## Discussion

In this study we utilized the NCC conditional deletion of *EdnrB* model of HSCR to systematically determine GI cellular immune defects that may contribute to the development of HAEC. In addition to recapitulating the clinical HSCR phenotype of distal colonic aganglionosis, our data indicate that loss of *EdnrB* specifically in the neural crest cells, and not in other cell types, is sufficient for development of the HAEC phenotype. We demonstrate that *EdnrB* ^**NCC-/-**^ animals display lymphopenia of their PP, namely a decrease in B220^+^IgM^+^IgD^high^ (mature) B-lymphocytes. These observations are found prior to histologic evidence of enterocolitis, suggesting a specific B-lymphocyte defect that may contribute to the development of enterocolitis. These animals also display a gut-specific reduction of SIgA, which may be secondary to the decreased mature B-lymphocytes population in the PP. We also determined that *EdnrB* ^**NCC-/-**^ demonstrate B-lymphocytopenia of the spleen, but display an increased proportion of mature B-lymphocytes and a paucity of B-lymphocytes within the marginal zones, observations suggesting possible defects in B-lymphocyte maturation and/or egress from the spleen. Finally, we noted equivalent proportion of circulating L-selectin+ Mature B lymphocytes in *EdnrB* ^**NCC+/-**^ and *EdnrB* ^**NCC-/-**^ animals, as well as equivalent levels of MAdCAM-1 in the PP, which suggests that circulating mature B-lymphocytes are capable of trafficking to the PP in these animals. Together, these data suggest that the spleen may be the primary anatomic site of B-lymphocyte defects in *EdnrB* ^**NCC-/-**^. Accordingly, these data are the first to demonstrate a cellular immune defect that may link the splenic immune apparatus and GI mucosal immunity in the context of HSCR.

Multiple lines of investigation point to interactions between the ENS and GI mucosal immune system. Anatomically, enteric neurons innervate the intestinal mucosa, including the gut-associated lymphoid tissue [51]. Enteric ganglia are positioned along the base, corona and interfollicular regions of PP [51–55]. Other investigators have demonstrated functional alterations in immune cell function associated with changes in the ENS, likely related to the presence of neuropeptide receptors on the surface of immune cells [56–58]. It has also been proposed that invasion of enteric nerves via the PP serves as a route of infection of the central nervous system by ingested prions [53,59].

The most common genetic defects associated with HSCR are mutations of *RET* and *EdnrB*, which are both required for NCC migration and ENS formation. *RET* has been identified as necessary for the development of PP [29]. In an elegant series of experiments [29,60], it has been shown that PP generation involves the aggregation of CD4+CD3−IL-7Ra+c-Kit+CD11c− lymphoid tissue inducer (LT_i_) cells, CD4−CD3−c-Kit+IL-7Ra−CD11c+RET+ lymphoid tissue initiator (LT_in_) cells, and mesenchymal lymphoid tissue organizer (LT_o_) cells, leading to formation of PP primordia in the midgut of mice by embryonic day 16.5 (E16.5). In the current study, we did not observe differences in the numbers of PP in *EdnrB* ^**NCC-/-**^ animals, but rather specific defects in B-lymphocyte populations within the PP and the spleen, suggesting perturbations in B-lymphocyte maturation, trafficking, or function.

Another group investigating the mechanisms of HAEC has noted reduced splenic size in *EdnrB* ^-/-^ animals [47]. They have additionally described abnormal splenic architecture and reduced total lymphocytes in the spleen. They postulate that this results in systemic immune deficiency that may predispose to HAEC. We noted similar findings of reduced splenic mass and altered architecture in our NCC-conditional *EdnrB* ^**NCC-/-**^, suggesting a role for NCC or *EdnrB* in lymphocyte development. Endothelin ligands (*ET-1*, *ET-3*) and receptors (*EdnrA*, *EdnrB*) form the endothelin axis, which participates in diverse biological processes including development, inflammation and cancer. The role of the endothelin axis in vascular biology has been well characterized [61]. However, beyond the vascular system, endothelins and their receptors are co-expressed in a wide variety of tissues and undergo coordinated changes in expression, suggesting a tightly-regulated, autocrine-paracrine mechanism of action [61,62]. There is extensive literature demonstrating modulation of lymphocyte function by endothelins [63–66]. Additionally, components of the endothelin axis are over-expressed in the serum and bowel wall of Crohn’s disease patients [67,68] and *EdnrA/B* antagonists can reduce inflammation in murine models of chemically-mediated colitis [69,70], underscoring a potential role for endothelins in bowel pathology. Indeed, we have previously described increased expression of *EdnrB* in the microenvironment of the *EdnrB* ^**NCC-/-**^ gut [37], suggesting that alterations in the endothelin axis outside of NCC may contribute to the phenotype. Further investigation into alterations of endothelin signaling outside the neural crest in our model may shed light on the HAEC phenotype.

B-lymphocytes targeted for mucosa-associated lymphoid tissue are identified by cell-surface integrins and their destination governed by chemokine/receptor interactions [71]. Mature B-cells bearing L-selectin and α_4_β_7_ integrin (L-selectin^high^α_4_β_7_^low^) leave the spleen and home to the PP, where they interact with MAdCAM-1 on the high endothelial venules of the PP through initial attachment (L-selectin) and rolling (α_4_β_7_) [72]. This homing is mediated by the chemokine CXCL13, expressed on the PP, and the receptor CXCR5, present on B-cells. Additionally, *ET-1*/*EdnrB* signaling mediates homing of T-cells to tumors by regulation of ICAM expression [73]. Therefore, while we did not observe differences in the circulating L-selectin^+^ B-lymphocyte population or PP MAdCAM-1 expression, it is possible that impaired chemokine or adhesion molecule signaling reduces trafficking of B-lymphocytes from the spleen to PP.

A mucus gel layer composed of several glycoproteins and secretory IgA (SIgA) lines the luminal border of the colon and provides a defensive barrier against pathogens and foreign particles. Multiple investigators have demonstrated alterations in subsets of mucins, a major component of this layer [15,74]. Additionally, the rate of mucin turnover in the bowel is decreased in HSCR patients [74]. Interestingly, both of these observations extend to the ganglionated as well as aganglionic regions of the bowel. Another group demonstrated an increase in IgA containing plasma cells along the length of resected aganglionic bowel from five patients with HAEC as compared to seven patients without HAEC [44]. They also demonstrated that luminal IgA (or SIgA) was reduced in the same patients, suggesting either a decrease in production or impaired transport to the lumen. We report here a similar observation of decreased luminal SIgA in the *EdnrB* ^**NCC-/-**^ animals, the first demonstration of this clinical correlate in a mouse model of HSCR/HAEC. This alteration in SIgA is accompanied by decreased PP mature B-lymphocytes, which serve as the primary source of murine SIgA. This suggests that the decreased number of B-lymphocytes may result in decreased SIgA production. However, in the current study we did not measure mucosa-associated IgA or lamina propria IgA^+^ plasma cells, which may be preserved and would indicate a defect in secretion, rather than production. Alternatively, our finding may be a reflection of the timing of our experiments, and that SIgA is being produced and secreted appropriately, but is rapidly bound to luminal antigens. Further functional experiments of *EdnrB* ^**NCC-/-**^ B-lymphocytes are warranted to distinguish these possibilities. Finally, we noted no differences in luminal IgM between *EdnrB* ^**NCC+/-**^ and *EdnrB* ^**NCC-/-**^ animals. An elevated level of IgM, produced primarily by lamina propria-resident B-lymphocytes, would have been unexpected because LP-resident B-lymphocytes do not typically make non-IgA isotypes until post-natal week 6 [46,75].

A notable limitation of our study is the presence of concomitant bowel obstruction secondary to distal colonic aganglionosis. The presence of the distal bowel obstruction may lead to stasis of intestinal contents, alterations in the microbiome, histologic changes, and sepsis. Certainly, previous investigations have demonstrated similar histological features in HAEC-affected bowel and obstructed bowel [14,15]. Additionally, similar changes in splenic lymphocyte populations can be found in response to sepsis/stress [76]. To dissect out the contribution of obstruction and stress to the immune phenotype of conventional *EdnrB* knockout animals, Frykman, et.al. have recently published a study in which they performed bone marrow transplants from *EdnrB* ^-/-^ animals to *Rag2* ^-/-^ recipients, as well as inducing bowel obstruction in WT animals [12]. In this study, they elegantly demonstrate that the stress from obstruction and the consequent elaboration of cortisone result in reductions in immune populations similar to those observed in our study and in their prior work. However, when their study is put in the context of their prior work, in which ~40% of *EdnrB* ^-/-^ animals develop HAEC after surgical relief of obstruction [9], one can conclude that relieving the bowel obstruction is not sufficient to prevent the development of HAEC. This result suggests that other factors contribute to the etiology of HAEC in the *EdnrB* ^-/-^ model. Indeed, in their recent study, Frykman, et.al. noted small splenic size in the *EdnrB* ^-/-^ bone marrow transplant recipients, concluding that *EdnrB* may play a role in lymphocyte trafficking between the bone marrow, thymus and spleen. Ultimately, our laboratory findings and those of other groups are in need of contemporary human tissue correlates in order to understand their true significance.

The increasingly challenged dogma in HSCR is that the abnormal bowel is limited to the aganglionic segment. Our data build upon the growing body of literature that suggests that intestinal defects in HSCR are not restricted to the aganglionic colon and extend proximally, even into the ganglionated small intestine. These abnormalities likely include subtle differences in extent of innervation and neuronal phenotypes, which may explain motility disturbances, as well as alterations in the microbiome composition and mucosal immune apparatus, which together may predispose to HAEC. Dissecting the mechanisms by which endothelin signaling participates in mucosal immune development will further our understanding of how the functions of the ENS and immune system are integrated and may provide insight into the development of HAEC.

## Supporting Information

S1 Fig T-lymphocyte populations in *EdnrB* ^NCC-/-^ PP. (A) The proportion of T-lymphocytes (CD3+) per PP is unchanged between *EdnrB* ^**NCC-/-**^ vs. *EdnrB* ^**NCC+/-**^. (B) The proportion of CD3+CD4+ and (C) CD3+CD8+ T-lymphocytes is unchanged between *EdnrB* ^**NCC-/-**^ vs.—het PP. (D) The number of T-lymphocytes per PP is decreased in *EdnrB* ^**NCC-/-**^ vs.—het PP (calculated values). (E) The number of CD3+CD4+ and (F) CD3+CD8+ T-lymphocytes is decreased in *EdnrB* ^**NCC-/-**^ vs.—het PP (calculated values).
(TIF)

## References

[1] Amiel J, Sproat-Emison E, Garcia-Barcelo M, Lantieri F, Burzynski G, Borrego S, et al Hirschsprung disease, associated syndromes and genetics: a review. J Med Genet. 2008;45: 1–14. 10.1136/jmg.2007.053959 17965226

[2] Pierre JF, Barlow-Anacker AJ, Erickson CS, Heneghan AF, Leverson GE, Dowd SE, et al Intestinal dysbiosis and bacterial enteroinvasion in a murine model of Hirschsprung's disease. J Pediatr Surg. 2014;49: 1242–1251. 10.1016/j.jpedsurg.2014.01.060 25092084 PMC4122863

[3] Sasselli V, Pachnis V, Burns AJ. The enteric nervous system. Dev Biol. 2012;366: 64–73. 10.1016/j.ydbio.2012.01.012 22290331

[4] Ward NL, Pieretti A, Dowd SE, Cox SB, Goldstein AM. Intestinal aganglionosis is associated with early and sustained disruption of the colonic microbiome. Neurogastroenterology & Motility. 2012;24: 874–e400. 10.1111/j.1365-2982.2012.01937.x 22626027

[5] Austin KM. The pathogenesis of Hirschsprung’s disease-associated enterocolitis. Semin Pediatr Surg. Elsevier Inc; 2012;21: 319–327. 10.1053/j.sempedsurg.2012.07.006 22985837

[6] Frykman PK, Short SS. Hirschsprung-associated enterocolitis: prevention and therapy. Semin Pediatr Surg. 2012;21: 328–335. 10.1053/j.sempedsurg.2012.07.007 22985838 PMC3462485

[7] Mundt E, Bates MD. Genetics of Hirschsprung disease and anorectal malformations. Semin Pediatr Surg. 2010;19: 107–117. 10.1053/j.sempedsurg.2009.11.015 20307847

[8] Gabriel SB, Salomon R, Pelet A, Angrist M, Amiel J, Fornage M, et al Segregation at three loci explains familial and population risk in Hirschsprung disease. Nat Genet. 2002;31: 89–93. 10.1038/ng868 11953745

[9] Sánchez-Mejías A, Fernández RM, López-Alonso M, Antiñolo G, Borrego S. New roles of EDNRB and EDN3 in the pathogenesis of Hirschsprung disease. Genet Med. 2010;12: 39–43. 10.1097/GIM.0b013e3181c371b0 20009762

[10] Kusafuka T, Wang Y, Puri P. Mutation analysis of the RET, the endothelin-B receptor, and the endothelin-3 genes in sporadic cases of Hirschsprung's disease. J Pediatr Surg. 1997;32: 501–504. 9094028 10.1016/s0022-3468(97)90616-3

[11] Kusafuka T, Wang Y, Puri P. Novel mutations of the endothelin-B receptor gene in isolated patients with Hirschsprung's disease. Human Molecular Genetics. 1996;5: 347–349. 8852658 10.1093/hmg/5.3.347

[12] Auricchio A, Casari G, Staiano A, Ballabio A. Endothelin-B receptor mutations in patients with isolated Hirschsprung disease from a non-inbred population. Human Molecular Genetics. 1996;5: 351–354. 8852659 10.1093/hmg/5.3.351

[13] Puffenberger EG, Hosoda K, Washington SS, Nakao K, deWit D, Yanagisawa M, et al A missense mutation of the endothelin-B receptor gene in multigenic Hirschsprung's disease. Cell. 1994;79: 1257–1266. 8001158 10.1016/0092-8674(94)90016-7

[14] Pini-Prato A, Rossi V, Avanzini S, Mattioli G, Disma N, Jasonni V. Hirschsprung's disease: what about mortality? Pediatr Surg Int. 2011;27: 473–478. 10.1007/s00383-010-2848-2 21253751

[15] Gershon MD. Developmental determinants of the independence and complexity of the enteric nervous system. Trends Neurosci. 2010;33: 446–456. 10.1016/j.tins.2010.06.002 20633936

[16] Best KE, Glinianaia SV, Bythell M, Rankin J. Hirschsprung's disease in the North of England: prevalence, associated anomalies, and survival. Birth Defects Res Part A Clin Mol Teratol. 2012;94: 477–480. 10.1002/bdra.23016 22511583

[17] Marty TL, Seo T, Matlak ME, Sullivan JJ, Black RE, Johnson DG. Gastrointestinal function after surgical correction of Hirschsprung's disease: long-term follow-up in 135 patients. J Pediatr Surg. 1995;30: 655–658. 7623220 10.1016/0022-3468(95)90682-7

[18] Veiga-Fernandes H, Coles MC, Foster KE, Patel A, Williams A, Natarajan D, et al Tyrosine kinase receptor RET is a key regulator of Peyer’s Patch organogenesis. Nature. 2007;446: 547–551. 10.1038/nature05597 17322904

[19] Polley TZ, Coran AG, Wesley JR. A ten-year experience with ninety-two cases of Hirschsprung's disease. Including sixty-seven consecutive endorectal pull-through procedures. Ann Surg. 1985;202: 349–355. 4037907 10.1097/00000658-198509000-00012 PMC1250915

[20] Zhao L, Dhall D, Cheng Z, Wang HL, Doherty TM, Bresee C, et al Murine model of Hirschsprung-associated enterocolitis II: Surgical correction of aganglionosis does not eliminate enterocolitis. J Pediatr Surg. 2010;45: 206–11; discussion 211–2. 10.1016/j.jpedsurg.2009.10.035 20105605 PMC4375950

[21] Lee C-C, Lien R, Chian M-C, Yang P-H, Chu S-M, Fu J-H, et al Clinical Impacts of Delayed Diagnosis of Hirschsprung’s Disease in Newborn Infants. Pediatrics and Neonatology. Elsevier Taiwan LLC; 2012;53: 133–137. 10.1016/j.pedneo.2012.01.011 22503261

[22] Brandtzaeg P. Mucosal immunity: induction, dissemination, and effector functions. Scand J Immunol. 2009;70: 505–515. 10.1111/j.1365-3083.2009.02319.x 19906191

[23] Murphy F, Puri P. New insights into the pathogenesis of Hirschsprung's associated enterocolitis. Pediatr Surg Int. 2005;21: 773–779. 10.1007/s00383-005-1551-1 16195910

[24] Frykman PK, Cheng Z, Wang X, Dhall D. Enterocolitis causes profound lymphoid depletion in endothelin receptor B- and endothelin 3-null mouse models of Hirschsprung-associated enterocolitis. Eur J Immunol. 2015;45: 807–817. 10.1002/eji.201444737 25487064 PMC4370321

[25] Schuchardt A, D'Agati V, Larsson-Blomberg L, Costantini F, Pachnis V. Defects in the kidney and enteric nervous system of mice lacking the tyrosine kinase receptor Ret. Nature. 1994;367: 380–383. 10.1038/367380a0 8114940

[26] Thiagarajah JR, Yildiz H, Carlson T, Thomas AR, Steiger C, Pieretti A, et al Altered goblet cell differentiation and surface mucus properties in Hirschsprung disease. PLoS ONE. 2014;9: e99944 10.1371/journal.pone.0099944 24945437 PMC4063789

[27] Bill AH, Chapman ND. The enterocolitis of Hirschsprung's disease: its natural history and treatment. Am J Surg. 1962;43: 70–73.

[28] Teitelbaum DH, Caniano DA, Qualman SJ. The pathophysiology of Hirschsprung's-associated enterocolitis: importance of histologic correlates. J Pediatr Surg. 1989;24: 1271–1277. 2593059 10.1016/s0022-3468(89)80566-4

[29] Bondurand N, Natarajan D, Barlow A, Thapar N, Pachnis V. Maintenance of mammalian enteric nervous system progenitors by SOX10 and endothelin 3 signalling. Development. 2006;133: 2075–2086. 10.1242/dev.02375 16624853

[30] Nagy N, Goldstein AM. Endothelin-3 regulates neural crest cell proliferation and differentiation in the hindgut enteric nervous system. Dev Biol. 2006;293: 203–217. 10.1016/j.ydbio.2006.01.032 16519884

[31] Glotzer DJ, Gpihl BG. Experimental obstructive colitis. Arch Surg. 1966;92: 1–8. 5901255 10.1001/archsurg.1966.01320190003001

[32] Hosoda K, Hammer RE, Richardson JA, Baynash AG, Cheung JC, Giaid A, et al Targeted and natural (piebald-lethal) mutations of endothelin-B receptor gene produce megacolon associated with spotted coat color in mice. Cell. 1994;79: 1267–1276. 8001159 10.1016/0092-8674(94)90017-5

[33] Baynash AG, Hosoda K, Giaid A, Richardson JA, Emoto N, Hammer RE, et al Interaction of endothelin-3 with endothelin-B receptor is essential for development of epidermal melanocytes and enteric neurons. Cell. 1994;79: 1277–1285. 8001160 10.1016/0092-8674(94)90018-3

[34] Shen D-H, Shi C-R, Chen J-J, Yu S-Y, Wu Y, Yan W-B. Detection of intestinal bifidobacteria and lactobacilli in patients with Hirschsprung's disease associated enterocolitis. World J Pediatr. 2009;5: 201–205. 10.1007/s12519-009-0038-x 19693464

[35] Gariepy CE, Williams SC, Richardson JA, Hammer RE, Yanagisawa M. Transgenic expression of the endothelin-B receptor prevents congenital intestinal aganglionosis in a rat model of Hirschsprung disease. J Clin Invest. 1998;102: 1092–1101. 10.1172/JCI3702 9739043 PMC509092

[36] De Filippo C, Pini-Prato A, Mattioli G, Avanzini S, Rapuzzi G, Cavalieri D, et al Genomics approach to the analysis of bacterial communities dynamics in Hirschsprung's disease-associated enterocolitis: a pilot study. Pediatr Surg Int. 2010;26: 465–471. 10.1007/s00383-010-2586-5 20306059

[37] Druckenbrod NR, Powers PA, Bartley CR, Walker JW, Epstein ML. Targeting of endothelin receptor-B to the neural crest. Genesis. 2008;46: 396–400. 10.1002/dvg.20415 18693272 PMC2610478

[38] Erickson CS, Zaitoun I, Haberman KM, Gosain A, Druckenbrod NR, Epstein ML. Sacral neural crest-derived cells enter the aganglionic colon of Ednrb(-/-) mice along extrinsic nerve fibers. J Comp Neurol. 2011 10.1002/cne.22755 PMC3500027 21858821

[39] Zaitoun I, Erickson CS, Barlow AJ, Klein TR, Heneghan AF, Pierre JF, et al Altered neuronal density and neurotransmitter expression in the ganglionated region of Ednrb null mice: implications for Hirschsprung's disease. Neurogastroenterol Motil. 2013 10.1111/nmo.12083 PMC3578114 23360229

[40] Cheng Z, Dhall D, Zhao L, Wang HL, Doherty TM, Bresee C, et al Murine model of Hirschsprung-associated enterocolitis. I: phenotypic characterization with development of a histopathologic grading system. J Pediatr Surg. 2010;45: 475–482. 10.1016/j.jpedsurg.2009.06.009 20223308 PMC4370315

[41] Fujimoto T, Reen DJ, Puri P. Inflammatory response in enterocolitis in the piebald lethal mouse model of Hirschsprung's disease. Pediatr Res. 1988;24: 152–155. 10.1203/00006450-198808000-00002 3054773

[42] Coles M, Kioussis D, Veiga-Fernandes H. Cellular and molecular requirements in lymph node and Peyer's patch development. Prog Mol Biol Transl Sci. 2010;92: 177–205. 10.1016/S1877-1173(10)92008-5 20800822

[43] Macpherson AJ, McCoy KD, Johansen F-E, Brandtzaeg P. The immune geography of IgA induction and function. Mucosal Immunology. 2008;1: 11–22. 10.1038/mi.2007.6 19079156

[44] Imamura A, Puri P, O'Briain DS, Reen DJ. Mucosal immune defence mechanisms in enterocolitis complicating Hirschsprung's disease. Gut. 1992;33: 801–806. 1624163 10.1136/gut.33.6.801 PMC1379340

[45] Rosado MM, Aranburu A, Capolunghi F, Giorda E, Cascioli S, Cenci F, et al From the fetal liver to spleen and gut: the highway to natural antibody. Mucosal Immunology. Nature Publishing Group; 2009;2: 351–361. 10.1038/mi.2009.15 19421184

[46] Harris NL, Spoerri I, Schopfer JF, Nembrini C, Merky P, Massacand J, et al Mechanisms of neonatal mucosal antibody protection. J Immunol. 2006;177: 6256–6262. 17056555 10.4049/jimmunol.177.9.6256

[47] Cheng Z, Wang X, Dhall D, Zhao L, Bresee C, Doherty TM, et al Splenic lymphopenia in the endothelin receptor B-null mouse: implications for Hirschsprung associated enterocolitis. Pediatr Surg Int. 2011;27: 145–150. 10.1007/s00383-010-2787-y 21046116 PMC3755962

[48] Vilagos B, Hoffmann M, Souabni A, Sun Q, Werner B, Medvedovic J, et al Essential role of EBF1 in the generation and function of distinct mature B cell types. Journal of Experimental Medicine. 2012;209: 775–792. 10.1084/jem.20112422 22473956 PMC3328360

[49] Berg EL, McEvoy LM, Berlin C, Bargatze RF, Butcher EC. L-selectin-mediated lymphocyte rolling on MAdCAM-1. Nature. 1993;366: 695–698. 10.1038/366695a0 7505053

[50] Loder F, Mutschler B, Ray RJ, Paige CJ, Sideras P, Torres R, et al B cell development in the spleen takes place in discrete steps and is determined by the quality of B cell receptor-derived signals. J Exp Med. 1999;190: 75–89. 10429672 10.1084/jem.190.1.75 PMC2195560

[51] Vulchanova L, Casey MA, Crabb GW, Kennedy WR, Brown DR. Anatomical evidence for enteric neuroimmune interactions in Peyer's patches. J Neuroimmunol. 2007;185: 64–74. 10.1016/j.jneuroim.2007.01.014 17363074 PMC1913558

[52] Krammer HJ, Kühnel W. Topography of the enteric nervous system in Peyer's patches of the porcine small intestine. Cell Tissue Res. 1993;272: 267–272. 8513481 10.1007/BF00302732

[53] Chiocchetti R, Mazzuoli G, Albanese V, Mazzoni M, Clavenzani P, Lalatta-Costerbosa G, et al Anatomical evidence for ileal Peyer's patches innervation by enteric nervous system: a potential route for prion neuroinvasion? Cell Tissue Res. 2008;332: 185–194. 10.1007/s00441-008-0583-y 18317812

[54] Balemba OB, Mbassa GK, Semuguruka WD, Assey RJ, Kahwa CK, Hay-Schmidt A, et al The topography, architecture and structure of the enteric nervous system in the jejunum and ileum of cattle. J Anat. 1999;195 (Pt 1): 1–9. 10473287 10.1046/j.1469-7580.1999.19510001.x PMC1467959

[55] Balemba OB, Grøndahl ML, Mbassa GK, Semuguruka WD, Hay-Smith A, Skadhauge E, et al The organisation of the enteric nervous system in the submucous and mucous layers of the small intestine of the pig studied by VIP and neurofilament protein immunohistochemistry. J Anat. 1998;192 (Pt 2): 257–267. 9643426 10.1046/j.1469-7580.1998.19220257.x PMC1467759

[56] Kaleczyc J, Podlasz P, Winnicka A, Wasowicz W, Sienkiewicz W, Zmudzki J, et al Characterization of Autonomic Nerve Markers and Lymphocyte Subsets in the Ileal Peyer's Patch of Pigs Infected Experimentally with Brachyspira hyodysenteriae. Journal of comparative pathology. 2010 10.1016/j.jcpa.2010.04.003 20605161

[57] Green BT, Lyte M, Kulkarni-Narla A, Brown DR. Neuromodulation of enteropathogen internalization in Peyer's patches from porcine jejunum. J Neuroimmunol. 2003;141: 74–82. 12965256 10.1016/s0165-5728(03)00225-x

[58] Pascual DW, Kiyono H, McGhee JR. The enteric nervous and immune systems: interactions for mucosal immunity and inflammation. Immunomethods. 1994;5: 56–72. 7531102 10.1006/immu.1994.1038

[59] Ano Y, Sakudo A, Nakayama H, Onodera T. Uptake and dynamics of infectious prion protein in the intestine. Protein Pept Lett. 2009;16: 247–255. 19275737 10.2174/092986609787601642

[60] Patel A, Harker N, Moreira-Santos L, Ferreira M, Alden K, Timmis J, et al Differential RET Signaling Pathways Drive Development of the Enteric Lymphoid and Nervous Systems. Science Signaling. 2012;5: ra55–ra55. 10.1126/scisignal.2002734 22855506

[61] Khimji A-K, Rockey DC. Endothelin—Biology and disease. Cellular Signalling. Elsevier B.V; 2010;22: 1615–1625. 10.1016/j.cellsig.2010.05.002 20466059

[62] Kandalaft LE, Facciabene A, Buckanovich RJ, Coukos G. Endothelin B Receptor, a New Target in Cancer Immune Therapy. Clinical Cancer Research. 2009;15: 4521–4528. 10.1158/1078-0432.CCR-08-0543 19567593 PMC2896814

[63] Yamaguchi E, Yamanoi A, Ono T, Nagasue N. Experimental investigation of the role of endothelin-1 in idiopathic portal hypertension. Journal of Gastroenterology and Hepatology. 2007;22: 1134–1140. 10.1111/j.1440-1746.2006.04822.x 17608860

[64] Sampaio AL, Rae GA, Henriques MG. Participation of endogenous endothelins in delayed eosinophil and neutrophil recruitment in mouse pleurisy. Inflamm Res. 2000;49: 170–176. 10858017 10.1007/s000110050577

[65] Malendowicz LK, Brelinska R, De Caro R, Trejer M, Nussdorfer GG. Endothelin-1, acting via the A receptor subtype, stimulates thymocyte proliferation in the rat. Life Sci. 1998;62: 1959–1963. 9619845 10.1016/s0024-3205(98)00165-9

[66] Guruli G. Function and survival of dendritic cells depend on endothelin-1 and endothelin receptor autocrine loops. Blood. 2004;104: 2107–2115. 10.1182/blood-2003-10-3559 15213100

[67] Letizia C, Boirivant M, De Toma G, Cerci S, Subioli S, Scuro L, et al Plasma levels of endothelin-1 in patients with Crohn's disease and ulcerative colitis. Ital J Gastroenterol Hepatol. 1998;30: 266–269. 9759593

[68] Murch SH, Braegger CP, Sessa WC, MacDonald TT. High endothelin-1 immunoreactivity in Crohn's disease and ulcerative colitis. The Lancet. 1992;339: 381–385. 1346658 10.1016/0140-6736(92)90077-g

[69] Hogaboam CM, Muller MJ, Collins SM, Hunt RH. An orally active non-selective endothelin receptor antagonist, bosentan, markedly reduces injury in a rat model of colitis. Eur J Pharmacol. 1996;309: 261–269. 8874149 10.1016/0014-2999(96)00276-2

[70] Anthoni C, Mennigen RB, Rijcken EJM, Laukötter MG, Spiegel H-U, Senninger N, et al Bosentan, an endothelin receptor antagonist, reduces leucocyte adhesion and inflammation in a murine model of inflammatory bowel disease. Int J Colorectal Dis. 2006;21: 409–418. 10.1007/s00384-005-0015-3 16088386

[71] Reese SR, Kudsk KA, Genton L, Ikeda S. l-selectin and alpha4beta7 integrin, but not ICAM-1, regulate lymphocyte distribution in gut-associated lymphoid tissue of mice. Surgery. 2005;137: 209–215. 10.1016/j.surg.2004.08.003 15674203

[72] Berlin C, Berg EL, Briskin MJ, Andrew DP, Kilshaw PJ, Holzmann B, et al Alpha 4 beta 7 integrin mediates lymphocyte binding to the mucosal vascular addressin MAdCAM-1. Cell. 1993;74: 185–195. 7687523 10.1016/0092-8674(93)90305-a

[73] Buckanovich RJ, Facciabene A, Kim S, Benencia F, Sasaroli D, Balint K, et al Endothelin B receptor mediates the endothelial barrier to T cell homing to tumors and disables immune therapy. Nature Medicine. 2008;14: 28–36. 10.1038/nm1699 18157142

[74] Aslam A, Spicer RD, Corfield AP. Children with Hirschsprung's disease have an abnormal colonic mucus defensive barrier independent of the bowel innervation status. J Pediatr Surg. 1997;32: 1206–1210. 9269971 10.1016/s0022-3468(97)90683-7

[75] Fagarasan S, Honjo T. Regulation of IgA synthesis at mucosal surfaces. Curr Opin Immunol. 2004;16: 277–283. 10.1016/j.coi.2004.03.005 15134775

[76] Toti P, De Felice C, Occhini R, Schuerfeld K, Stumpo M, Epistolato MC, et al Spleen depletion in neonatal sepsis and chorioamnionitis. Am J Clin Pathol. 2004;122: 765–771. 10.1309/RV6E-9BMC-9954-A2WU 15491973

