# Supplementary figures and images for: Impaired Cellular Immunity in the Murine Neural Crest Conditional Deletion of Endothelin Receptor-B Model of Hirschsprung’s Disease
Source: PLoS One. 2015 Jun 10;10(6):e0128822. doi: 10.1371/journal.pone.0128822 (PMC4465674; doi:10.1371/journal.pone.0128822)

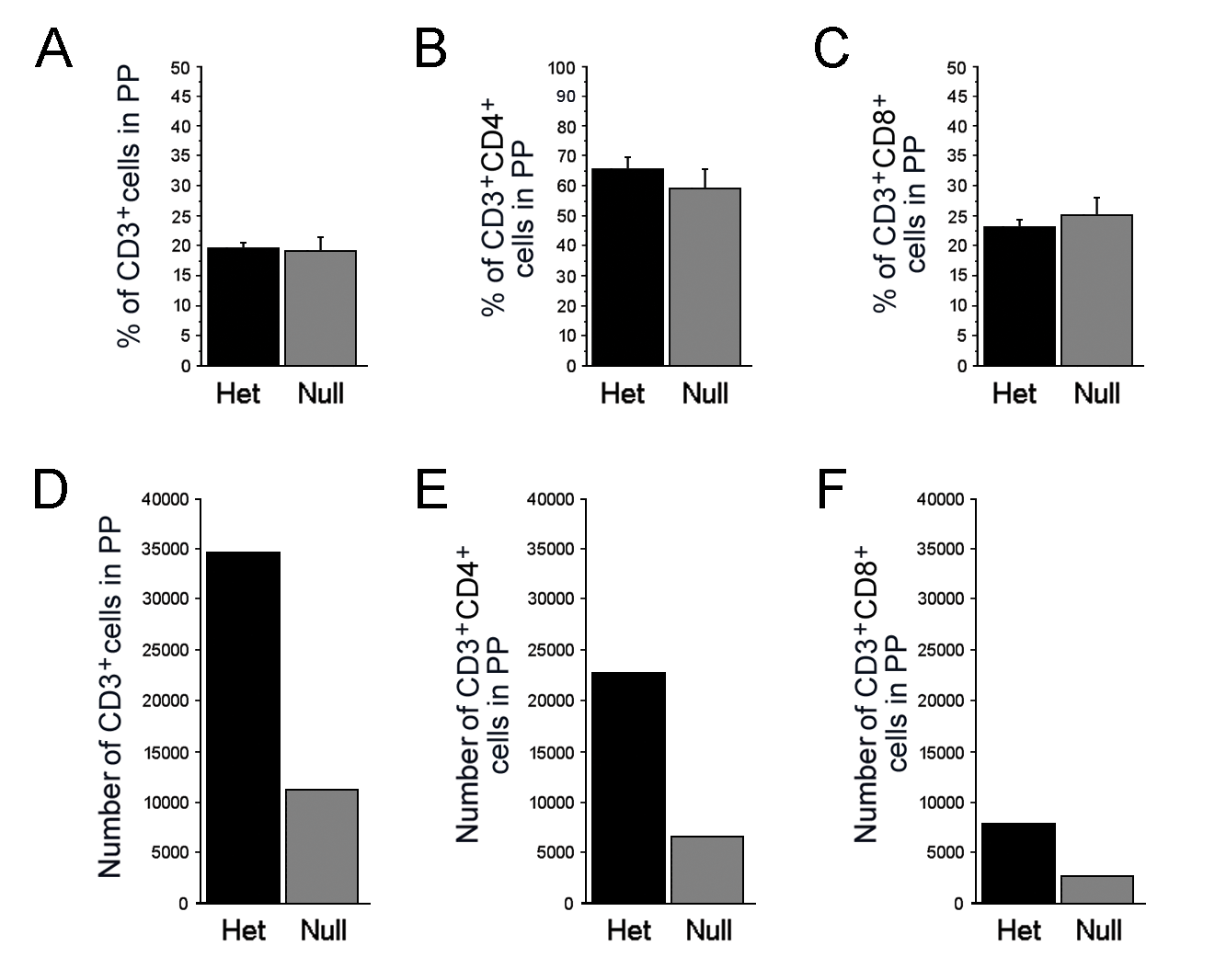

Supplement: S1 Fig — (A) The proportion of T-lymphocytes (CD3+) per PP is unchanged between EdnrB NCC-/- vs. EdnrB NCC+/-. (B) The proportion of CD3+CD4+ and (C) CD3+CD8+ T-lymphocytes is unchanged between EdnrB NCC-/- vs.—het PP. (D) The number of T-lymphocytes per PP is decreased in EdnrB NCC-/- vs.—het PP (calculated values). (E) The number of CD3+CD4+ and (F) CD3+CD8+ T-lymphocytes is decreased in EdnrB NCC-/- vs.—het PP (calculated values). (TIF) [file pone.0128822.s001.tif]
